# Indoor use of attractive toxic sugar bait in combination with long-lasting insecticidal net against pyrethroid-resistant *Anopheles gambiae*: an experimental hut trial in Mbé, central Côte d’Ivoire

**DOI:** 10.1186/s12936-019-3095-1

**Published:** 2020-01-07

**Authors:** Joanna E. C. Furnival-Adams, Soromane Camara, Mark Rowland, Alphonsine A. Koffi, Ludovic P. Ahoua Alou, Welbeck A. Oumbouke, Raphael N’Guessan

**Affiliations:** 1 London School of Hygiene and Tropical Medicine, Keppel Street, London, UK; 2 Vector Control Product Evaluation Centre (VCPEC), Institut Pierre Richet, Bouaké, Côte d’Ivoire

**Keywords:** Attractive toxic sugar bait, *Anopheles gambiae*, Sugar feeding behaviour, Resistance management

## Abstract

**Background:**

Indoor attractive toxic sugar bait (ATSB) has potential as a supplementary vector-control and resistance-management tool, offering an alternative mode of insecticide delivery to current core vector-control interventions, with potential to deliver novel insecticides. Given the high long-lasting insecticidal bed net (LLIN) coverage across Africa, it is crucial that the efficacy of indoor ATSB in combination with LLINs is established before it is considered for wider use in public health.

**Methods:**

An experimental hut trial to evaluate the efficacy of indoor ATSB traps treated with 4% boric acid (BA ATSB) or 1% chlorfenapyr (CFP ATSB) in combination with untreated nets or LLINs (holed or intact), took place at the M’bé field station in central Côte d’Ivoire against pyrethroid resistant *Anopheles gambiae* sensu lato.

**Results:**

The addition of ATSB to LLINs increased the mortality rates of wild pyrethroid-resistant *An. gambiae* from 19% with LLIN alone to 28% with added BA ATSB and to 39% with added CFP ATSB (p < 0.001). *Anopheles gambiae* mortality with combined ATSB and untreated net was similar to that of combined ATSB and LLIN regardless of which insecticide was used in the ATSB. The presence of holes in the LLIN did not significantly affect ATSB-induced *An. gambiae* mortality. Comparative tests against pyrethroid resistant and susceptible strains using oral application of ATSB treated with pyrethroid demonstrated 66% higher survival rate among pyrethroid-resistant mosquitoes.

**Conclusion:**

Indoor ATSB traps in combination with LLINs enhanced the control of pyrethroid-resistant *An. gambiae*. However, many host-seeking *An. gambiae* entering experimental huts with indoor ATSB exited into the verandah trap without sugar feeding when restricted from a host by a LLIN. Although ATSB has potential for making effective use of classes of insecticide otherwise unsuited to vector control, it does not exempt potential selection of resistance via this route.

## Background

Malaria remains a major public health problem globally, with around 93% of malarial deaths occurring in Africa, despite substantial gains being achieved in the past through indoor residual spraying (IRS) and long-lasting insecticidal nets (LLINs) [1, 2]. Whilst vector control is an effective strategy for malaria control, there is increasing evidence to suggest that insecticide resistance is reducing the effectiveness of current control measures, and progress is beginning to stall in parts of sub-Saharan Africa [1, 3, 4]. To combat the effects of pyrethroid resistance and to prevent further development of resistance, alternative tools and combination strategies specific to the local context should be implemented. The potential for indoor attractive toxic sugar bait (ATSB) to target an alternative point in the mosquito life cycle and to deliver novel insecticides makes it a promising candidate as a supplementary malaria vector control tool. If efficacious, its deployment indoors would disproportionately target older female mosquitoes, with the highest risk of being infective.

ATSB takes advantage of the natural sugar feeding behaviour in female adult mosquitoes, using a sugar source as an attractant and killing the mosquito upon ingestion [5]. Sugar feeding occurs in both male and female mosquitoes. In females it influences the success of energy-demanding activities such as mating, host-seeking, blood feeding, development, and egg-laying [6]. Although evidence suggests that sugar-feeding in female *Anopheles gambiae* is relatively uncommon when blood hosts are readily available, mosquitoes are more likely to feed on sugar when energy is deficient [6–8]. For this reason, increasing restriction of blood hosts likely increases the diversion of mosquitoes to sugar sources, and the combination of ATSB and bed nets (at high coverage) may be synergistic.

There have been several successful attempts to control mosquito populations using ATSB outdoors [5, 9, 10]. ATSB for indoor use, however, has not been studied as extensively, although some trials have yielded promising results [11, 12]. Whilst outdoor ATSB targets residual outdoor transmission, indoor ATSB targets host-seeking endophagic, endophilic, pyrethroid-resistant mosquitoes that have survived exposure to pyrethroid-treated LLIN or IRS. A semi-field trial demonstrated that indoor ATSB in combination with an untreated bed net can induce notable levels of *Anopheles arabiensis* mortality in Tanzania [11]. A larger study conducted in Mali showed that the introduction of indoor ATSB bait stations to a hamlet of 19 houses led to a significant reduction in the local mosquito population [12]. These results are very promising; however, the local availability of insecticide-treated nets (ITNs) in this trial was not mentioned. Considering that LLINs constitute the standard of preventive care in Africa, it is important to gather further evidence on the efficacy of ATSB traps in combination with pyrethroid-treated LLINs before wider indoor deployment can be considered. The uncertainty lies in whether mosquitoes will be diverted towards the ATSB stations after failing to blood feed through a LLIN, or whether the repellent effect of the LLIN will cause host-seeking mosquitoes to exit huts before taking the opportunity to feed on ATSB.

In addition to the efficacy of indoor ATSB against pyrethroid-resistant mosquitoes, the ability of pyrethroid-resistant *Anopheles gambiae* sensu lato (*s.l*.) (resistant to tarsal contact with pyrethroid) to resist pyrethroid delivered in the form of ATSB was assessed. Recent evidence has shown that mosquitoes can succumb to insecticide administered through one mode of delivery that they would resist when administered through another mode [13]. Pyrethroid delivery via electrostatic netting coated with powder formulation, for example, will kill mosquitoes that would resist pyrethroid delivered by LLINs and tarsal contact. ATSB constitutes a novel means of deploying insecticide against mosquitoes in which the toxin is administered by ingestion and absorption through the midgut. It is, therefore, possible that mosquitoes resistant to insecticide through tarsal contact (as observed in relation to the current core vector-control strategies, LLINs and IRS) would withstand insecticide delivered as ATSB via the midgut, i.e., whether ATSB would select for resistance.

The main aim of this study was to assess under quasi-household conditions the benefit of adding indoor ATSB to LLINs against pyrethroid-resistant *An. gambiae.* Bioassays using insecticide resistant and susceptible strains assessed whether insecticide ingested as ATSB via the midgut would show the same toxicity effect as does contact with LLIN or IRS interventions.

## Methods

### Mosquito strains and collection for laboratory experiments

Adults *An. gambiae s.l.* (M’bé strain) emerged from larvae collected in rice fields adjacent to the trial site were resistant to pyrethroids, DDT and carbamates. Underlying mechanisms include multi-function oxidases, esterases and point mutations [14]. *Anopheles gambiae* (Kisumu strain) susceptible strain originated from Kenya.

### Preparation of ATSB solution and bioassays for putative dosage determination

The attractive sugar bait (ASB) solution was based on a recipe of 35% guava juice purchased locally from a supermarket, 10% sugar solution, 2% orange food dye also bought locally in the same supermarket. The ATSB solution also contained boric acid concentrations of 0.2–2% and chlorfenapyr concentrations of 0.05–0.5% as the toxin [11]. Guava juice is known to be a strong attractant for *An. gambiae* [5]. The ATSB (juice + toxicant) was prepared afresh each morning and the juice was not fermented prior to use in the field.

Boric acid sourced from Sigma-Aldrich and chlorfenapyr from BASF SE, both from Germany, were chosen for use in ATSB. Boric acid is an inorganic poison, widely used in agriculture, known to induce mortality in mosquitoes within 48 h of contact [15]. Chlorfenapyr is a pro-insecticide, activated by cytochrome P450s within the insect, and is transferred via tarsal contact or midgut passage [16]. Chlorfenapyr shows no cross resistance to common insecticide classes, is effective against pyrethroid-resistant mosquitoes, and has resistance management potential [17, 18]. Both insecticides were shown to be effective in ATSB in previous studies [11].

Bioassays were performed on 3–5 days old *An. gambiae* Kisumu (susceptible) and M’bé (resistant) using the exact ATSB solution (25 ml) that avoids excess dripping when soaked onto cotton wool pad. The bait was placed on a petri dish on the floor of the mosquito-rearing cage (40 cm^3^) and exchanged on a daily basis. Mosquitoes were starved for 6 h prior to the laboratory experiments by removing the sugar sources from the testing cages. Serial concentrations were tested: 0.2, 0.5, 1, 2% boric acid and 0.05, 0.125, 0.25, 0.5% chlorfenapyr, and 25 ml of ASB (without insecticide) was used as a control. Mosquitoes were left overnight to interact with the ATSB then scored in the morning for immediate mortality. Live mosquitoes were supplied with cotton pad soaked in 10% sucrose solution in observation cages and mortality recorded up to 72 h due to the potential slow toxic actions of the insecticides. The metal cages, including the netting material for wrap up were renewed before every bioassay replicate to avoid any sugar carry over and contamination.

### ATSB potential to select for insecticide resistance

An advantage of ATSB is that it brings novel, systemic insecticides to malaria vector control. The question of whether this would ultimately select for insecticide resistance was investigated by comparing the differential survival of pyrethroid-resistant and susceptible mosquitoes. 0.5% deltamethrin was incorporated into the ATSB and baited bioassays were performed on *An. gambiae* Kisumu (susceptible) and M’bé (resistant) strains using soaked cotton wool pads inserted into testing cages. Mortality was measured the next morning, live mosquitoes were supplied with 10% sucrose solution and mortality monitored 24 h later. Control mosquitoes were exposed to ASB solution without deltamethrin.

### Experimental hut trial

The experimental hut trial was carried out from 29 June to 7 September, 2018 at the M’bé field site, Bouaké, Côte d’Ivoire. The site is located next to rice fields, which provided extensive breeding sites for mosquitoes. The local community was provided with universal coverage of LLINs. Bouaké is characterized by wet savannah climate and a rainy season from April to October. The local mosquito fauna was comprised of *An. gambiae s.l., Anopheles funestus, Culex* spp. and *Mansonia* spp. [19]. *Anopheles coluzzii* and *An. gambiae* co‐exist but *An. coluzzii* represents 98% of the total *An. gambiae s.l.* [19].

**Fig. 1:**
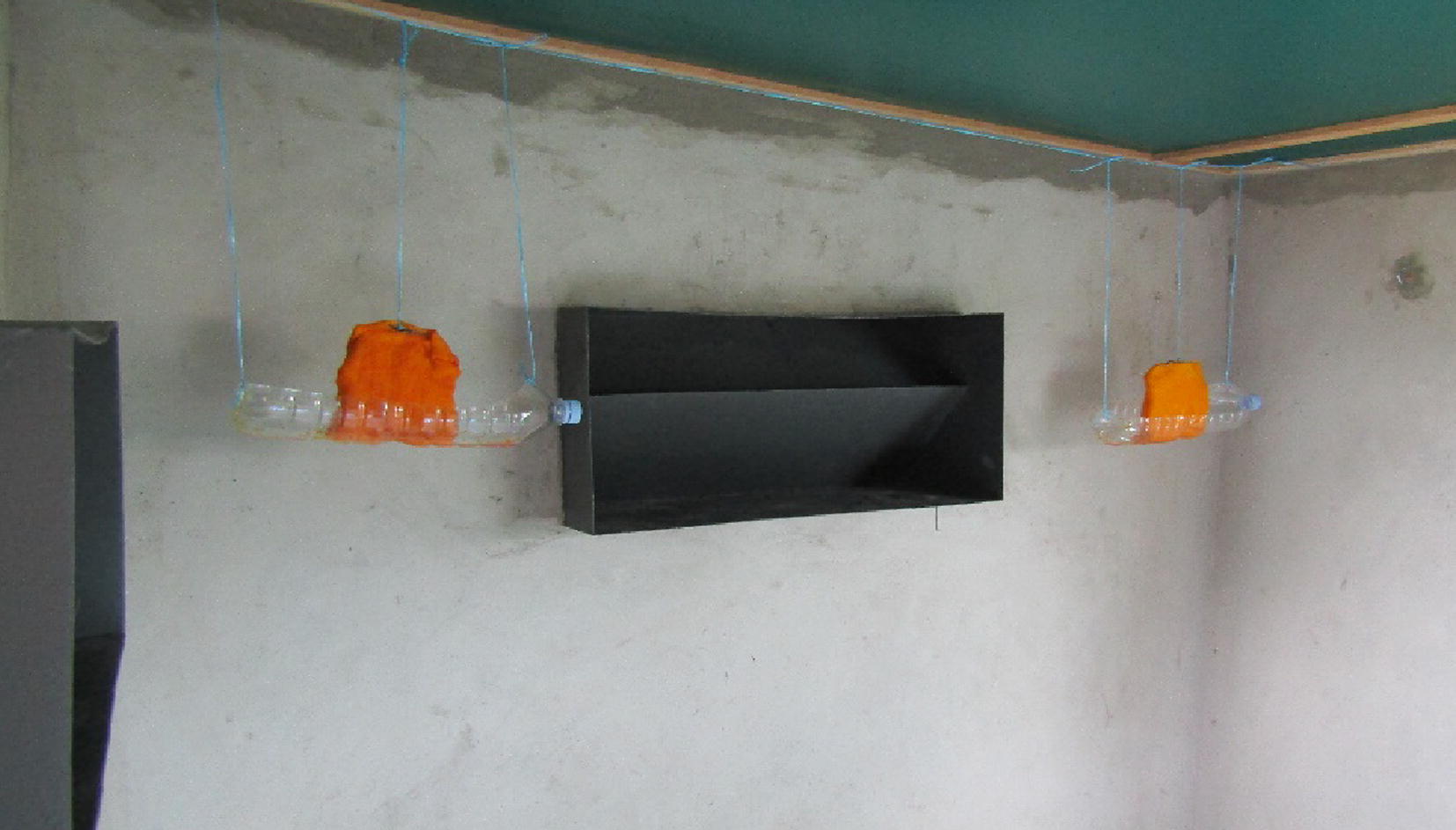
ATSB trap design and positioning inside experimental huts

A suite of six Western African style huts [20] were used for the study. The huts with size 3 m × 2 m were made of concrete bricks, with corrugated iron roofs and concrete plinths. Four window slits (1 cm wide) in the walls allowed unidirectional mosquito entry. A veranda trap on one of the walls collected exiting mosquitoes. A moat surrounding each hut excluded scavenging ants. A mattress and bed net were positioned in the centre of the hut. Four ATSB traps were positioned in the vicinity of the windows (Fig. 1). Treatment arms were rotated on a weekly basis using a Latin square design to adjust for variation in attractiveness between huts.

The ATSB traps were made using 20 cm × 30 cm cotton wool pads soaked in 250 ml ATSB solution. The saturated pads were wrapped around a frame and hung over a reservoir (made from half a plastic water bottle) to catch the excess and provide a wick (Fig. 1). The traps were replenished every 2 days with fresh ATSB solution and pads before any fermentation was observed.

### Study design

Twice the minimum lethal dosage determined in laboratory experiments was applied as the field rate in the huts: 4% boric acid ATSB (BA) and 1% chlorfenapyr ATSB (CFP). PermaNet 2.0 LN in two treatment conditions were tested: one holed to simulate a worn net (P 2.0 LNh) and one intact (P 2.0 LNi). An untreated net also holed was included. Six holes (4 cm × 4 cm) were made in both holed nets and all nets were unwashed.

The following arms were included in the trial:

1. Untreated polyester net holed (UNh).
2. PermaNet 2.0 LN holed (P2.0 LNh).
3. Boric acid 4% ATSB + untreated polyester net holed (BA + UNh).
4. Boric acid 4% ATSB +PermaNet 2.0 LN holed (BA + P2.0 LNh).
5. Chlorfenapyr 1% ATSB + untreated polyester net holed (CFP + UNh).
6. Chlorfenapyr 1% ATSB + PermaNet 2.0 LN holed (CFP + P2.0 LNh).
7. Chlorfenapyr 1% ATSB + PermaNet 2.0 LN intact (CFP + P2.0 LNi).

Volunteer male sleepers slept in the huts between 19:00 and 06:00, 6 nights per week and rotated to a different hut each night to adjust for differences in attractiveness between individuals. Arms 1–6 of the trial took place over a 6-week period. The objective of arms 1–6 was to assess the efficacy of indoor ATSB traps in combination with holed untreated nets and LLINs. Arm 7 (CFP + P2.0 LNi) was started 4 weeks into the trial together with a new control arm, a holed untreated net (UN), which also lasted for 6 weeks. The objective of this additional arm was to assess whether in the presence of a chemical barrier with an intact LLIN, mosquitoes are more likely to feed on ATSB.

**Fig. 2:**
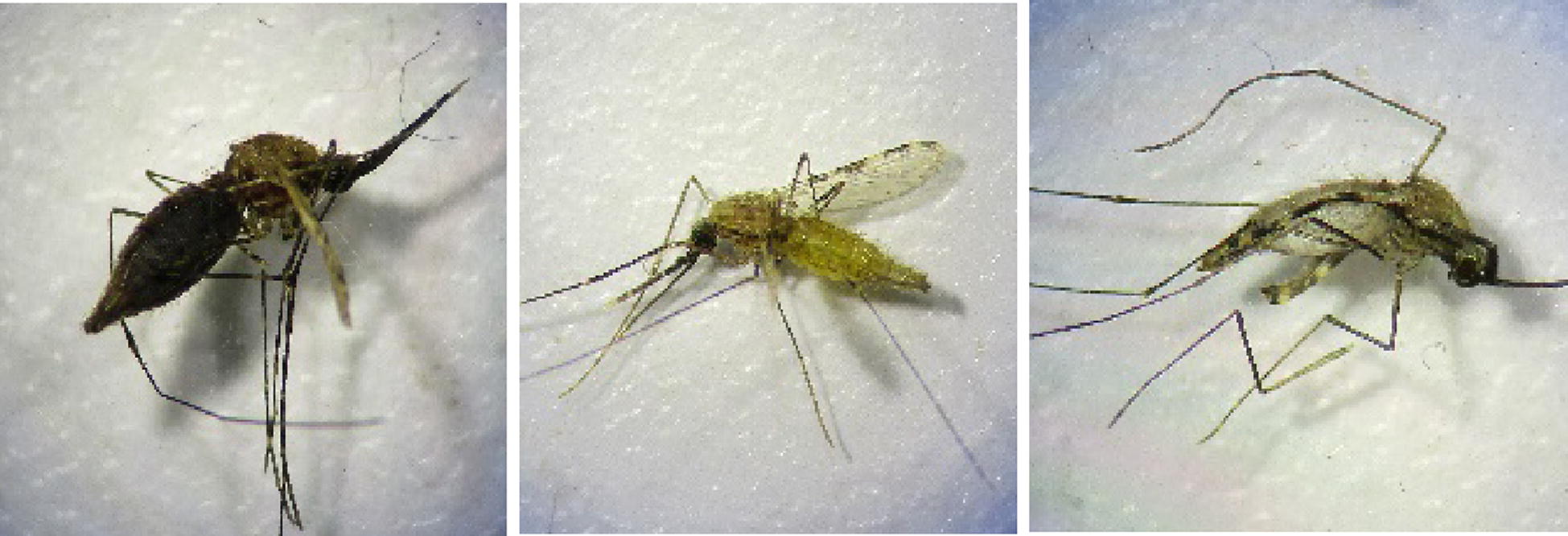
The appearance of blood-fed (left), ATSB fed (middle) and sugar-fed (right) mosquitoes under the microscope

Mosquitoes were collected from under the net, on the walls and roof and from the veranda of the huts every morning at 06:00 with an aspirator. Females were recorded as blood fed, ATSB fed or unfed and identified to species (Fig. 2). Orange food dye was added to the ATSB trap pad to allow ATSB fed mosquitoes to be distinguished visually from blood-fed and sugar-fed mosquitoes. Live mosquitoes were held with sugar solution and delayed mortality recorded up to 72 h.

The primary outcomes measured were:

- Deterrence: the proportional reduction in hut entry relative to entry in the control hut (with an untreated net only;
- Exophily: the proportion of mosquitoes found in the veranda traps;
- Blood-feeding inhibition: the proportional reduction in blood feeding relative to blood feeding observed in the control hut;
- ATSB-feeding: proportion of mosquitoes with food dye found in their abdomen (that was visible under the microscope);
- Personal protection: the proportional reduction in the number of blood-fed mosquitoes collected in the huts with ATSB treatments relative to the hut with untreated net only, as per the formula 100(B_u_ − B_t_)/B_u_
  where Bu = total number blood-fed mosquitoes in the control hut; and Bt = total number blood-fed mosquitoes in the huts with treatments;
- Mortality at collection: the proportion of mosquitoes found dead in the morning of collection;
- 72-hour mortality: the proportion of mosquitoes found dead 24, 48 and 72 h after collection.

### Tunnel tests

The tunnel test is an overnight insecticide bioassay of host-seeking mosquitoes that mimics conditions around nets in experimental huts [20]. The tunnel is a rectangular glass cylinder (height 25 cm, width 21 cm, length 60 cm) divided into two chambers by framed netting insert which is punctured by 9 × 1 cm diameter holes. At the baited end of the tunnel a pigeon was held in a cage. In the mosquito release chamber, the ATSB, a cotton wick containing 25 ml (2% boric acid or 0.5% chlorfenapyr solution was placed near the entrance. Approximately 100 unfed female mosquitoes (5–7 days old) were introduced in the release chamber at 18:00 and the apparatus was left overnight in a dark room maintained at 26 °C ± 2 °C and 80% ± 2% relative humidity. The next morning, at 08.00, the mosquitoes in both compartments were counted and mortality, blood‐feeding and ATSB-feeding status were scored. Live mosquitoes were transferred to a plastic cup, supplied with 10% sucrose solution, and mortality was scored after 24, 48 and 72 h. Tests were replicated two or three times for each treatment. The following treatments were compared:

1. Untreated net (holed).
2. P2.0 LNh (holed).
3. Boric acid (2%) + untreated net (holed).
4. Boric acid (2%) + P2.0 LNh (holed).
5. Chlorfenapyr (0.5%) + untreated net (holed).
6. Chlorfenapyr (0.5%) + P2.0 LNh (holed).

### Statistical analysis

All data were entered into Excel and then transferred to R (version 3.5.1) for further analysis. The experimental hut data were analysed using the “lme4” package to perform logistic regression for proportional data (blood feeding inhibition, induced mortality, exophily) and adjusted for clustering of mosquitoes by day; these results were used to compare the results from different treatment arms of the experiment. The number of collected mosquitoes entering the huts (deterrence) and the actual number of blood-fed mosquitoes (personal protection) were analysed using negative binomial regression using the “glmmADMB” package.

Data from the tunnel tests and bioassays were analysed using Z-tests for proportional data and bar charts were plotted in R using “ggplot2”.

## Results

### ATSB feeding bioassays to determine field putative dosage for boric acid and chlorfenapyr

**Fig. 3:**
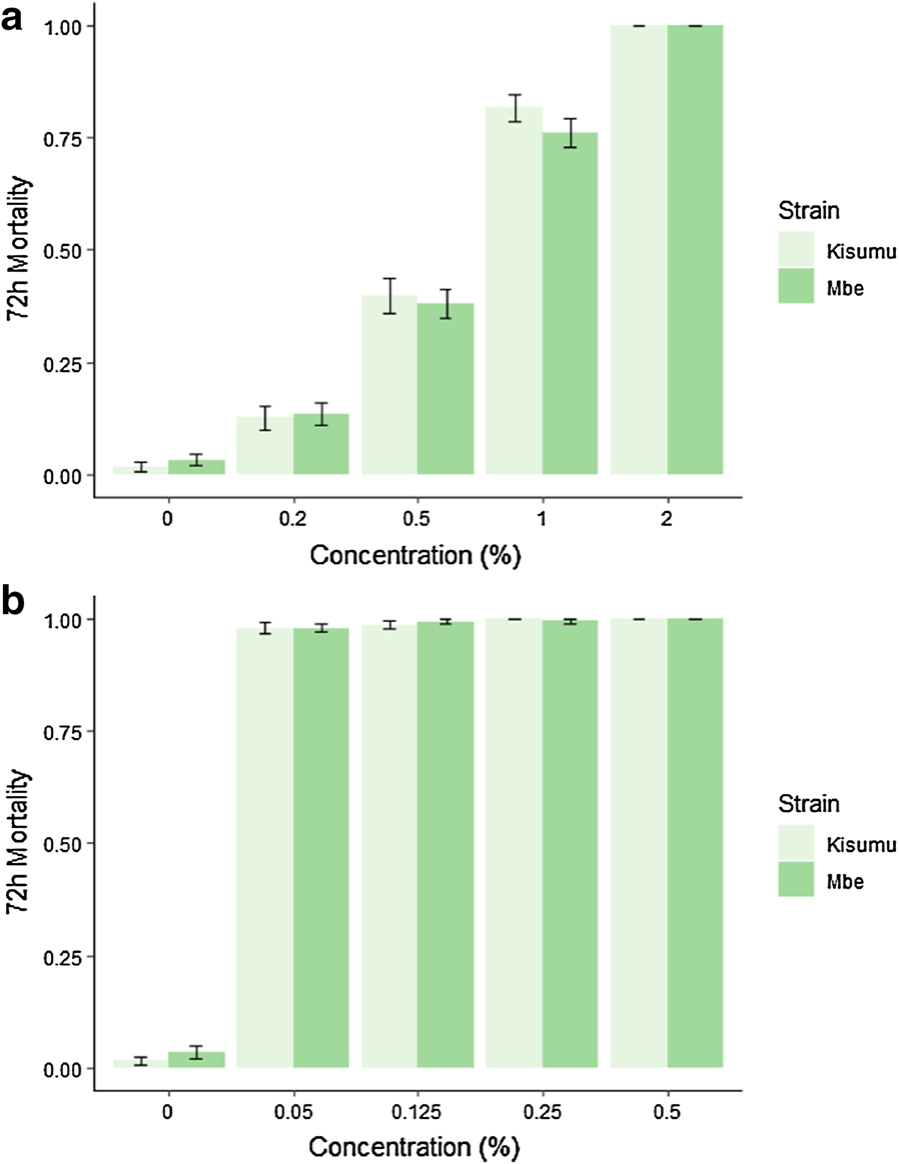
Proportions of *Anopheles gambiae s.l.* killed after 72 h in preliminary cage bioassays to determine putative dosages for boric acid (**a**) and chlorfenapyr (**b**)

In the preliminary bioassays to establish the optimal field concentration of ATSB, the higher the boric acid concentration the higher the mortality, indicating a clear dose-dependent effect with this insecticide (Fig. 3a). Concentrations of 2% boric acid and 0.5% chlorfenapyr were required to kill 100% of both strains (Fig. 3b). Mortality of M’bé strain did not differ from that of the Kisumu strain for either of the insecticides nor for any of the dosages tested, suggesting there was no cross resistance to these insecticides present in the field strain. Twice the putative dosage for each insecticide (4% boric acid and 1% chlorfenapyr) was selected as the field concentration.

### Experimental hut trial

Table 1 reports the numbers entering and proportions exiting the experimental huts. The numbers of mosquitoes entering each control hut were comparable: 752 in control 1 (weeks 1–6) and 626 in control 2 (weeks 4–10) and therefore data from the two controls were pooled. Most treatments induced significant deterrence relative to the untreated net reference arm. The exception was the boric acid + untreated net arm. The LLIN made most of the contribution to deterrence. Significantly fewer mosquitoes were caught in the CFP + untreated net arm (p < 0.001).

**Table 1:** Effect of ATSB (boric acid BA, chlorfenapyr CFP) with untreated net or PermaNet 2.0 (P2.0) LN on the numbers of *Anopheles gambiae s.l.* caught: percentages deterred and percentages exiting in experimental huts

Treatment	Total females caught	Mean number of females caught per night	Percentage deterred	Percentage exiting
Untreated net^a^	745	23^a^ (17–30)	─	40^ac^ (36–44)
P2.0 LN (holed)	403	12^bc^ (9–16)	49	50^b^ (44–55)
BA + untreated net (holed)	628	17^ab^ (13–23)	25	37^a^ (33–42)
BA + P2.0 LN (holed)	451	12^bc^ (9–16)	48	45^bc^ (40–50)
CFP + untreated net (holed)	508	14^b^ (11–19)	39	33^a^ (28–38)
CFP + P2.0 LN (holed)	462	12^bc^ (9–17)	45	47^b^ (42–53)
CFP + P2.0 LN (intact)	326	8.8^c^ (6.5–12)	61	60^d^ (54–66)

Numbers in the same column sharing a letter superscript do not differ significantly (p > 0.05)

^a^Pooled average of the two control huts (described in *“*Methods*”*)

P 2.0 LNh induced significantly greater exiting of *An. gambiae s.l.* compared to untreated nets, either alone or combined with ATSB (p < 0.001). The exiting rate associated with ATSB in the presence of untreated net was not significantly different from the exiting observed with untreated net alone. The combination of CFP + intact PermaNet 2.0 induced higher exiting than the other treatments.

Table 2 summarizes the effect of the treatment arms on blood feeding and ATSB feeding (indicated by the presence of food dye in the abdomen). Each treatment arm that included LLIN showed significantly greater blood-feeding inhibition compared to the respective arm, which included an untreated net, irrespective of ATSB presence. The presence of boric acid ATSB did not appear to affect blood feeding over the effect of the net. By contrast, the blood-feeding rates associated with CFP ATSB + P2.0 LNh (26%) or CFP ATSB + P2.0 LNi (19%) were significantly less than with the P2.0 LNh alone (35%) (Table 2). Blood-feeding inhibition with the CFP + P2.0 LNh treatment was almost twice that of the BA + P2.0 LNh treatment (p = 0.02). Blood-feeding rate in the CFP ATSB + P2.0 LNi arm was significantly lower than in the CFP ATSB + P2.0 LNh arm.

**Table 2:** Effect of ATSB (Boric acid BA, chlorfenapyr CFP) with untreated net or PermaNet 2.0 (P2.0) LN on the proportions of *Anopheles gambiae s.l.* blood fed and ATSB fed (visible dye in abdomen) in experimental huts

Treatment	Total blood fed	Percentage blood fed	BFI (%)	Personal protection (%)	Females with visible dye in abdomen (%)
Untreated net^a^	331	46^a^ (41–51)	─	─	─
P2.0 LN (holed)	138	35^b^ (30–41)	24	64	─
BA + untreated net (holed)	268	42^a^ (37–48)	8	31	8^a^ (6–10)
BA + P2.0 LN (holed)	151	33^b^ (28–39)	28	62	8^a^ (5–10)
CFP + untreated net (holed)	242	47^a^ (41–52)	0	37	6^ac^ (4–8)
CFP + P2.0 LN (holed)	119	26^c^ (21–31)	44	70	11^b^ (8–14)
CFP + P2.0 LN (intact)	61	19^d^ (14–24)	60	84	4^c^ (2–7)

Numbers in the same column sharing the same letter superscript do not differ significantly (p > 0.05)

*BFI* Blood feeding inhibition, *NS* not statistically significant (p > 0.05)

^a^Pooled average of the two control huts (described in *“*Methods*”*)

ATSB feeding, as indicated by the presence of food dye in mosquito abdomens, ranged from 4 to 12% in the ASTB arms, and did not reflect the mortality associated with ATSB (Table 2), suggesting that the food dye used was an unreliable indicator of ATSB ingestion.

Table 3 summarizes the mortality associated with each treatment. Percentage mortality of *An. gambiae* in the LLIN arm was significantly higher than the mortality observed with the untreated reference net (p < 0.001). The pyrethroid-induced mortality was less than 10% due to resistance. ATSB containing BA or CFP toxicants combined with untreated nets induced significantly higher levels of *An. gambiae* mortality than the pyrethroid LN alone (p < 0.001). The presence of the LN in combination with BA or CFP ATSB had no additional effect on mortality compared to the combination of ATSB and untreated net.

**Table 3:** Effect of ATSB (Boric acid BA, chlorfenapyr CFP) with untreated net or PermaNet 2.0 (P2.0) LN on *Anopheles gambiae s.l.* mortality in experimental huts

Treatment	Total dead after 72 h	Percentage mortality at collection	Percentage mortality after 24 h	Percentage mortality after 72 h	Control-corrected 72 h mortality (%)	Total with visible dye	(%) Visible dye-fed mortality
Untreated net^a^	72	7^a^ (5–9)	10^a^ (8–12)	11^a^ (9–13)	─	0	─
LN (holed)	76	12^b^ (9–15)	17^b^ (13–21)	19^b^ (15–23)	9	0	─
BA + untreated net (holed)	178	19^c^ (16–23)	25^c^ (21–29)	28^c^ (24–32)	19	51	94^a^ (92–96)
BA + P2.0 LN (holed)	124	23^c^ (19–28)	27^c^ (22–31)	28^c^ (23–33)	19	35	94^a^ (92–96)
CFP + untreated net (holed)	189	34^d^ (29–39)	36^d^ (31–41)	37^d^ (32–42)	29	29	100
CFP + P2.0 LN (holed)	181	33^d^ (28–38)	37^d^ (32–42)	39^d^ (34–44)	31	53	100
CFP + P2.0 LN (intact)	109	18^c^ (14–23)	27^c^ (22–33)	33^cd^ (28–39)	25	14	100

Numbers in the same column sharing a letter superscript do not differ significantly (p > 0.05)

^a^Pooled average of the two control huts (described in “Methods”)

Whether combined with a LN or untreated net, mortality with CFP ATSB was significantly higher than that with BA ATSB (p < 0.001). The presence or absence of holes in the LN did not appear to influence percentage mortality: an intact LN with CFP ATSB showed a percentage mortality not significantly different to that of holed LN with CFP ATSB.

Mortality among blood-fed mosquitoes was 9% with holed LN alone. With the ATSB-holed LN combinations it was 13%; only a few per cent higher than LN alone (p > 0.05) (Table 4). This indicates that host-seeking mosquitoes that succeed in blood feeding show little or no interest in the ATSB after blood feeding. With respect to non-blood-fed mosquitoes, the mortality rate was 24% in the arm with holed LN alone and ranged from 30 to 49% with the BA and CFP ATSB-holed LN combinations and from 40 to 57% with the BA and CFP ATSB-holed untreated net arms (Table 4). This additional mortality among non-blood-fed mosquitoes can be attributable to ATSB; it was significantly greater in the presence of untreated nets than with LN (p < 0.001). Higher proportions of mosquitoes exited into the verandahs in the ATSB-LN arms than in ATSB-untreated net arms (Table 1); host-seeking mosquitoes which contact the LN first may be repelled by the pyrethroid and exit without sugar feeding whereas mosquitoes which contact untreated nets first may proceed to feed on ATSB and die in greater proportions (Table 4).

**Table 4:** Effect of ATSB (boric acid BA, chlorfenapyr CFP) with untreated net or PermaNet 2.0 (P2.0) LN on mortality of blood-fed and unfed *Anopheles gambiae s.l.* in experimental huts

Treatment	Total females blood fed dead	% dead among blood fed (blood fed dead/total blood fed)	Control corrected blood fed dead (%)	Total females unfed	% females unfed (total unfed/total caught)	% dead among unfed (unfed dead/total unfed)	Control corrected unfed dead (%)
Untreated net^a^	9	3^a^ (2–4)	─	393	56^a^ (52–61)	16^a^ (13–18)	─
LN (holed)	13	9^b^ (7–12)	6	267	66^b^ (60–72)	24^b^ (21–28)	10
BA + untreated net (holed)	32	11^b^ (9–14)	8	359	59^a^ (54–65)	40^c^ (36–45)	29
BA + LN (holed)	19	13^bc^ (10–16)	10	304	69^bc^ (63–74)	30^d^ (26–34)	17
CFP + untreated net (holed)	36	15^c^ (12–18)	13	264	55^a^ (49–61)	57^e^ (34–52)	48
CFP + LN (holed)	14	13^bc^ (10–16)	11	358	75^c^ (70–80)	49^f^ (44–53)	29
CFP + LN (intact)	14	24^d^ (19–28)	22	256	84^d^ (79–88)	34 ^g^ (30–39)	22

Numbers in the same column sharing a letter superscript do not differ significantly (p > 0.05)

^a^Pooled average of the two control huts (described in *“*Methods*”*)

### Tunnel tests

**Fig. 4:**
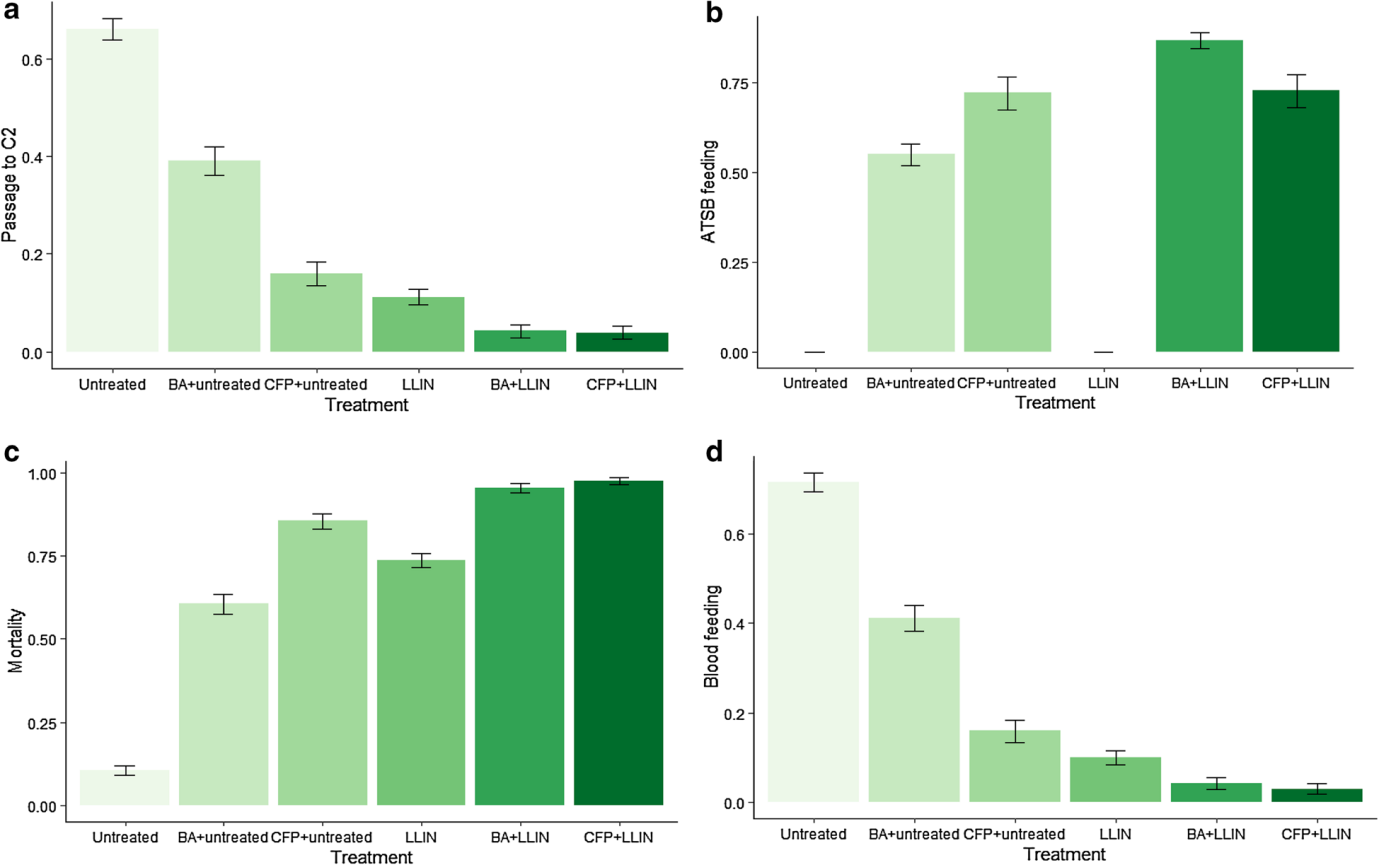
Effect of ATSB with untreated net or PermaNet 2.0 LN against pyrethroid-resistant *Anopheles gambiae s.l.* in tunnel test: (**a**) passage rate; (**b**) percentage ATSB feeding; (**c**) percentage 72-h mortality; (**d**) percentage blood feeding

The trend in passage rates between treatments mirrored the trend in blood feeding (Fig. 4a, d). Reduced penetration and blood feeding were mostly associated with the LN. Whilst 65.9% of mosquitoes penetrated the holed untreated netting, only 11.2% penetrated the holed LN (p < 0.001). There was a significant reduction in passage when ATSB was added to the untreated net: 39% passage with boric acid (p < 0.001) and 16% with chlorfenapyr (p < 0.001). The difference in netting penetration rate between BA ATSB and CFP ATSB with untreated netting was significant (p < 0.001). This is likely due to the more immediate and potent effects of chlorfenapyr on mortality in tunnel tests; mosquitoes that fed on ATSB before reaching the net likely died or were incapacitated before they had the chance to progress through the tunnel. The combination of the LN with ATSB reduced further the passage rate to 4.2% for the BA ATSB (p < 0.001) and to 3.9% for the CFP ATSB (p < 0.001).

The blood-feeding rate was highest in the untreated control (72%). The addition of ATSB significantly reduced blood feeding, with a more pronounced effect for chlorfenapyr than for boric acid (p < 0.001). Combining the LN with ATSB further reduced the blood feeding of *An. gambiae* relative to the P2.0 LN only arm (p = 0.015 for BA + P2.0 LN and p = 0.003 for CFP + 2.0 LN) and to the ATSB + untreated net arms (p < 0.001 for boric acid and p < 0.001 for chlorfenapyr).

Between 75 and 100% of mosquitoes that penetrated the netting went on to blood feed. In CFP arms, 70–71% mosquitoes fed on ATSB and in BA arms 85–91% fed on ATSB (Fig. 4b). This suggests that > 50% fed on ATSB before attempting to penetrate the net, and the toxic effect of BA and CFP inhibited any attempt to penetrate the net. This is where the tunnel test differs from the hut in which the host-seeking mosquito encountered and attempted to penetrate the net before approaching the ATSB near the window.

Mortality in the control arm (untreated net) was 10.6%. The mortality rate of the M’bé strain with the P2.0 LN-only arm was 65% (Fig. 4c). Mortality rate with ATSB and untreated net exceeded 50% and presumably intoxication impaired mosquito capacity to penetrate the netting and blood-feed. The combination of ATSB and P2.0 LN was additive, and significantly increased the mortality for both ATSBs compared to ATSB with untreated netting (p < 0.001). Very high mortality rates were observed for the ATSB and P2.0 LN combinations.

### Cross-resistance between tarsal contact and ingestion of pyrethroid

In the ATSB-feeding bioassays with 0.5% deltamethrin there was 100% mortality with the susceptible Kisumu strain and 34.2% mortality with the pyrethroid-resistant M’bé strain within 24 h of exposure. Resistance to deltamethrin in M’bé strain was demonstrated in a recent study, where WHO susceptibility tests with 0.05% deltamethrin test papers showed 75.8% mortality with M’bé and 100% mortality with Kisumu [14]. This indicates resistance is expressed with both methods of insecticide delivery (tarsal contact and ingestion), and that ATSB delivery has the potential to select for heritable resistance.

## Discussion

Although most studies investigating ATSB as a vector control tool have focussed on outdoor use, indoor ATSB has potential to target host-seeking mosquitoes provided they are used alongside LLINs. A previous experimental hut trial in Tanzania showed that indoor ATSB traps are effective against *An. arabiensis* when combined with untreated, intact nets [11]. Despite increasing evidence that pyrethroid resistance is diminishing the epidemiological gains from LLINs [4, 21], they are still recommended for use due to the netting barrier and the exito-repellency that provides personal protection, and the other sub-lethal effects that may contribute to malaria control [1]. For this reason, it is crucial that indoor ATSB traps effectively control mosquito populations in combination with LLINs before they can be considered for indoor use as a public health intervention. The present field trial was amongst the first to investigate the use of indoor ATSB traps in combination with LLINs against pyrethroid-resistant *An. gambiae.* The key question addressed by this study was whether host-seeking mosquitoes are repelled or inhibited from feeding on the ATSB on contact with a holed or intact pyrethroid-treated net or exit the hut before having the opportunity to feed on ATSB.

The mortality achieved in the experimental huts against pyrethroid-resistant mosquitoes from the indoor ATSB traps demonstrates their potential to mitigate the effects of pyrethroid resistance through the incorporation of non-pyrethroid insecticides. The mortality rate observed in the LLIN-only hut was comparatively low at 17%, which was expected in this area due to the high levels of pyrethroid resistance [14]. The presence of an ATSB trap significantly increased this mortality rate by about 10% for boric acid and 20% for chlorfenapyr when combined with holed untreated net or LLIN, respectively. After correcting for control mortality these mortality rates are about 6% lower than the rates observed in experimental huts in Tanzania in 2013, where the same ATSB solutions were used [11]. For indoor ATSB to work effectively, further optimization of the bait will be important in the future. The presence of a strong attractant is particularly important in an indoor and outdoor environment where the sugar source (ATSB) is competing with a blood host. The capacity of ATSB traps to incorporate novel insecticides that can kill pyrethroid-resistant mosquitoes in the presence of an LLIN makes them a promising candidate as an insecticide resistance management tool.

Whilst all of the ATSB treatments increased *An. gambiae* mortality compared to the LLIN-only treatment, mortality rates in huts with ATSB plus untreated net were almost identical with ATSB plus LLIN; this was true for both BA and CFP ATSB. This means that the LLIN and ATSB were not additive in their effect on mortality. A comparison between mortality patterns in blood-fed and unfed mosquitoes shed light on the behaviour that may be underlying this observation (Table 4).

The presence or absence of holes in the LLIN appeared to have no effect on ATSB-induced mosquito mortality. Although this is positive in the sense that the efficacy of ATSB is not reduced when the net is holed, it also reveals important information regarding the behaviour of mosquitoes that enter the hut. Prior to the trial, it was hypothesized that host-seeking *An. gambiae* would behave selectively with respect to their choice of meal, and that when deprived of a blood host, they would feed opportunistically on sugar to replenish their energy reserves. Whilst this model may still hold, the lack of selective feeding on ATSB in this study would suggest that the host-seeking mosquitoes that enter the hut did not always feed on sugar after failing to blood feed and may choose to remain unfed for another night before being diverted to a sugar source. It is unclear what the fate of the unfed mosquitoes that leave the hut would be. Ultimately, they would likely turn to sugar bait in another sugar-baited house if denied a blood meal by another net barrier. Although sugar feeding is facultative for the majority of female *An. gambiae*, it becomes a critical source of energy when these mosquitoes are deprived of blood [7]. The proportion of mosquitoes that fed on the ASTB after failing to blood feed on the sleeper was below 40% and this may reflect the proportion of mosquitoes feeding on sugar per single house visit. This may represent the proportion of mosquitoes that are most energy deprived among those first entering the hut. It is also possible that the type or concentration of the attractant (Guava juice here) in the ATSB is limited. Further studies are needed to screen and identify suitable attractants for ATSB.

Supplementary to the experimental hut trial, tunnel tests were conducted to investigate the effect of ATSB traps on host-seeking mosquitoes in a laboratory simulator. The tunnel test results differed from the experimental hut and indicated that the combination of ATSB and LLIN would increase mortality rates compared to ATSB and untreated net. This inconsistency may be due to the release of laboratory-reared mosquitoes that were not exclusively host seeking and may have been more inclined to feed on sugar compared to the mosquitoes entering the experimental huts. A 3-chamber tunnel design where the mosquitoes move up an odour concentration gradient to an ATSB-baited middle chamber and from there through treated netting to the host-baited third chamber might have produced results more consistent with the experimental huts tests.

Expression, dominance and selection of insecticide resistance is affected by the mode of delivery of the insecticide. An aerosol droplet of pyrethroid may deliver a sudden high dose of insecticide that kills a resistant, flying insect which a pre-sprayed surface would merely repel [22]. Novel insecticide delivery method through electrostatic coating of netting with pyrethroid in eave tubes has been shown to induce much higher killing rate of pyrethroid-resistant mosquitoes than pyrethroid LLINs [13]. It was proposed that insecticide delivery via the midgut rather than the less penetrable cuticle of the exo-skeleton would be more amenable to concentration of insecticide in the soft tissues of the mosquito and less likely to select for resistance. The question considered as part of this study was whether pyrethroid-resistant mosquitoes would exhibit the same level of resistance against ingested pyrethroids through the medium of ATSB compared to absorption via pyrethroid-treated nets. By using a different delivery system, it was hoped to increase the bio-availability of the insecticide and break resistance mechanisms. However, the present results showed that there was significantly less mortality of the resistant M’bé strain that ingested pyrethroid compared to the susceptible Kisumu strain, demonstrating that ATSB with pyrethroid as a delivery system should not be expected to overcome pyrethroid resistance in highly resistant strain of *An. gambiae* nor assumed to have pyrethroid resistance-breaking potential. It does remain an excellent delivery system for a broad range of non-pyrethroid insecticides which were originally designed for ingestion by chewing or sucking agricultural pest insects.

A limitation of this study was the lack of a reliable indicator of ATSB feeding. The difference observed in all treatment arms between mortality attributable to ATSB feeding and overall corrected mortality was substantial. However, only a small proportion of mosquitoes had visibly dyed abdomens. Given this, it can confidently stated that food dye used was not a sensitive measurement of ATSB feeding. Whilst the food dye was clearly visible when the mosquito had fed extensively on ATSB (Fig. 2), it is likely that some of the mosquitoes ingested an amount of ATSB that was undetectable under the microscope. This is likely for chlorfenapyr, which was shown to kill high proportions of mosquitoes at low concentrations (Fig. 3). It is also possible that the ATSB in the abdomens of mosquitoes that fed early in the evening before the collections would have desiccated prior to collection and was no longer visible under the microscope.

Key challenges for the implementation of indoor ATSB at scale will be developing ATSB stations that are durable and easy to maintain by household users and improving the attractiveness of the bait to unfed mosquitoes that have failed to blood feed through a LLIN. For example, fermenting the bait over days may be more attractive to mosquitoes, resulting in increased impact through increased feeding on the ‘ageing bait’. This study was a proof of concept; it was a precursor to research and development into applicator design. Any ATSB design which has to be replenished regularly may increase costs and be vulnerable to user fatigue and compliance issues [23]. A sustainable ATSB design should identify a way to improve bait preservation and enhance the design to make it more acceptable to householders long-term.

## Conclusion

This study provides evidence that indoor ATSB traps are effective in targeting pyrethroid-resistant *An. gambiae s.l.* and increasing mortality when combined with LLINs. The prospect for the combination of indoor ATSB traps and LLINs to reduce pyrethroid-resistant mosquito populations, whilst maintaining personal protection gained from the LLIN, is encouraging. However, the study revealed that the majority of mosquitoes that entered the hut were not immediately diverted to ATSB when thwarted from host blood feeding. Host-seeking mosquitoes that fail to blood feed may remain unfed for a further night. On exiting the house if they continue to fail to find a blood meal source they may ultimately feed on an ATSB when energy reserves are sufficiently diminished.

## Data Availability

All data generated or analysed during this study are included in this published article.

## Abbreviations

ATSB: attractive toxic sugar bait
BA: boric acid
CFP: chlorfenapyr
LLIN: long lasting insecticidal net
IRS: indoor residual spraying
